# Supplementary material for: Rod-cone signal interference in the retina shapes perception in primates
Source: Front Ophthalmol (Lausanne). 2023 Jul 31;3:1230084. doi: 10.3389/fopht.2023.1230084 (PMC11182321; doi:10.3389/fopht.2023.1230084)
Supplement: Supplementary file 1 [file DataSheet_1.pdf]

Supplementary Material

Rod-cone signal interference in the retina shapes perception in primates

Adree Songco-Aguas, William N Grimes, and Fred Rieke*

*** Correspondence:** Fred Rieke: rieke@uw.edu

Supplementary Figures

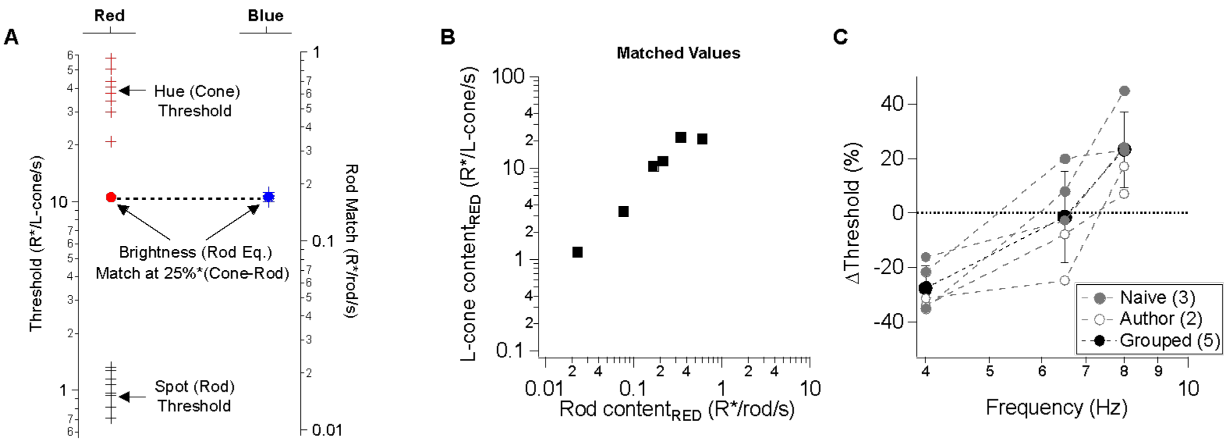

Supplementary Figure 1 (related to Figure 1). Additional details pertaining to the psychophysical experiments. A) To improve the selectivity of the L-cone-preferring stimulus, we obtained rod-equivalent matches for each observer under scotopic conditions and utilized the results to create silent-substitution under mesopic conditions (see Methods). An observer first finds the detection threshold for a static long wavelength spot. Next the observer finds a hue threshold for the same spot. Lastly, the long wavelength spot is fixed at 25% of the difference between the average hue and detection thresholds and another short wavelength spot is adjusted until a brightness match is achieved. B) Rod-equivalent matches for all observers. Plot depicts the empirically measured rod content of the long wavelength spot. C) Changes in flicker thresholds for combined stimulus presentations for each observer (i.e. breakdown of Figure 1C).

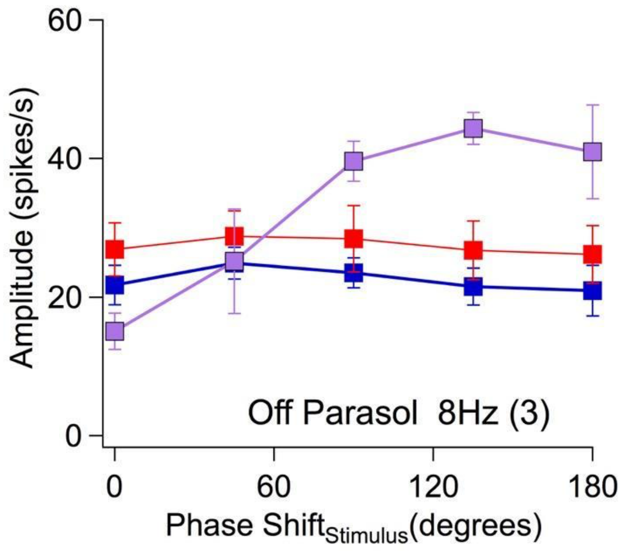

Supplementary Figure 2 (related to Figure 3): Rod-cone signals constructively and destructively interfere within Off Parasol RGC circuits. At 8 Hz, rod and cone signals can be destructive or constructive depending on the relative delay between the rod and cone stimuli.

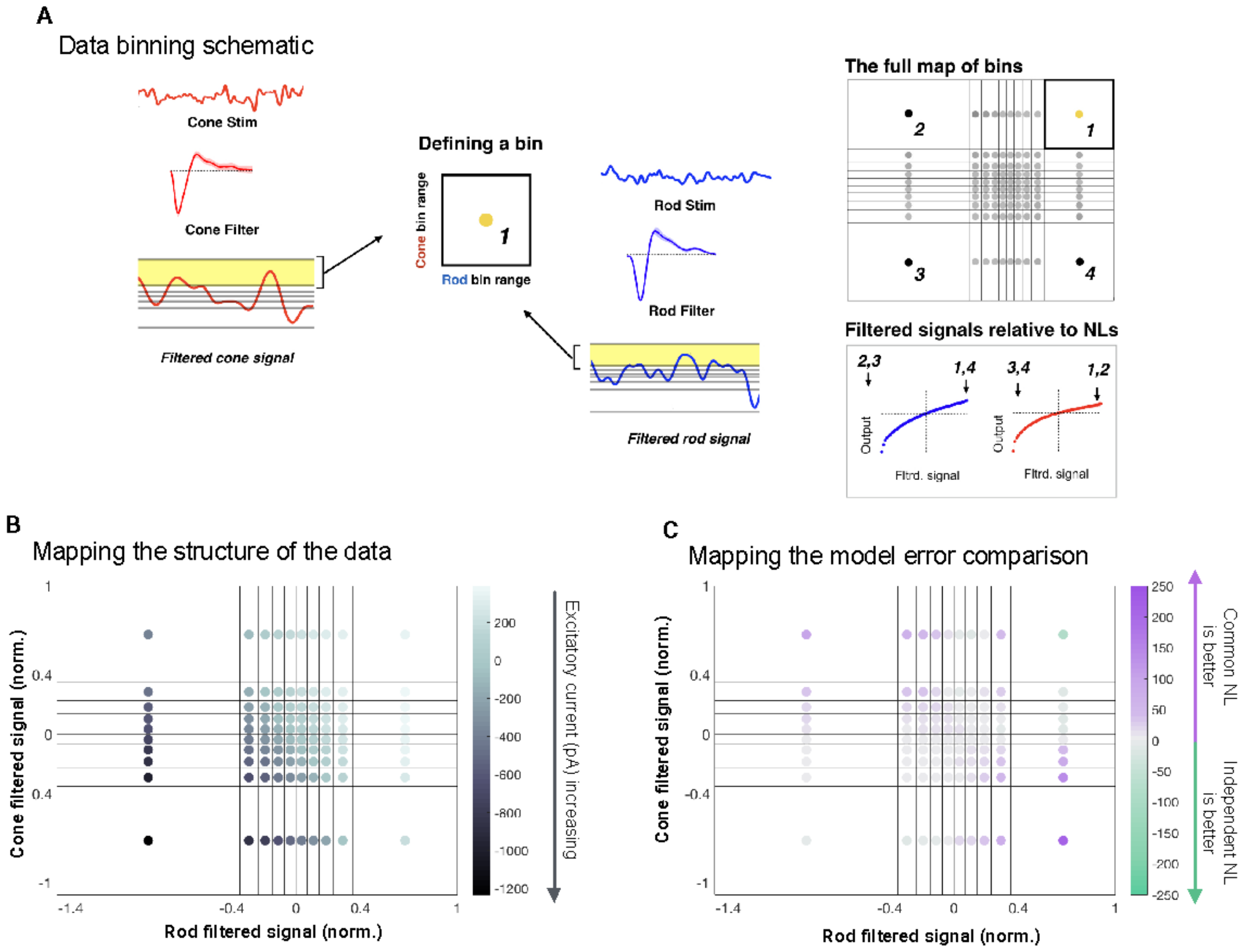

Supplementary Figure 3 (related to Figure 6): Dependence of model performance on specific rod-cone contributions to the signal. A) Regardless of the model architecture (e.g. common or independent nonlinearities), the initial linear filtering stage is separate for the rod and cone branches of the model. Hence, we sort the data based on bins based on the rod and cone filtered signals. The bins are equally populated with data points across five cells. A) A diagram representing the horizontal and vertical ranges of each bin (note: diagram does not depict real bin divisions). B) In plotting the mean current value across single bins, we see a clear underlying structure (i.e. excitatory current input increases as both filtered signals become both larger and more negative). This provides assurance that the binning method cleanly captures the data. C) The error between a model and the data in a single bin is taken as the absolute difference between the mean data and the mean model output. We compare the errors of the two models by simply subtracting the common nonlinearity error from the independent nonlinearity error (as the independent model had the larger average error). The common nonlinearity model outperforms the independent model in nearly every case where there is a difference in performance difference between the two models; in particular, this improvement was largest along the diagonal where the rod and cone filtered stimuli are the most anti-correlated.
